# Epidemiology and Healthcare Service Utilization among Adults with Chronic Cough

**DOI:** 10.3390/jcm13113230

**Published:** 2024-05-30

**Authors:** Gabriel Chodick, Yael Barer, Tal Blay Hagai, Ido Keidar, Gally Rosenfeld Teper, Hagit Kopel, Neville Berkman

**Affiliations:** 1 Maccabitech, Maccabi Institute for Research and Innovation, Maccabi Healthcare Services, Ha’Mered St. 27, Tel Aviv 6812509, Israel; chodick@tauex.tau.ac.il; 2 Sackler School of Medicine, Tel Aviv University, P.O. Box 39040, Ramat Aviv, Tel Aviv 6997801, Israel; 3 Global Medical and Scientific Affairs, Merck Sharp & Dohme Company Ltd., Ha’Charash St. 34, P.O. Box 7340, Hod Hasharon 45800, Israel; tal.hagay@merck.com (T.B.H.); idokeidar@gmail.com (I.K.); gally.teper@merck.com (G.R.T.); hagitkopel@gmail.com (H.K.); 4 Department of Pulmonary Medicine, Hadassah Medical Center and Faculty of Medicine, Hebrew University of Jerusalem, Kalman Ya’Akov Man Street, Ein-Karem, Jerusalem 9112102, Israel; neville@hadassah.org.il

**Keywords:** chronic cough, epidemiology, prevalence, healthcare service utilization, economic burden

## Abstract

**Background and objective:** Chronic cough (CC) is a prevalent yet underexplored medical condition, with limited real-world data regarding its healthcare burden. This study investigates the epidemiology, associated comorbidities, and healthcare service utilization among patients with CC. **Methods:** In this retrospective cohort study, adult patients with at least 3 physician diagnoses of cough over a period spanning a minimum of 8 weeks and a maximum of 12 months anytime between 2009 and 2018, were defined as patients with CC (PwCC). The reference group were adults without cough matched in a 1:1 ratio for age, sex, and place of residence. **Results:** The study included 91,757 PwCC, reflecting a prevalence of 5.5%. Of those, 59,296 patients (mean [SD] age, 53.9 [16.8] years; 59.6% females) were first diagnosed with CC during the study period, representing a 10-year incidence rate of 3.26% (95%CI: 3.24–3.29%). Diseases associated with the highest OR for CC included lung cancer (OR = 3.32; 95%CI: 2.90–4.25), whooping cough (OR = 3.04; 95%CI: 2.70–3.60), and respiratory infections (OR = 2.81; 95%CI: 2.74–2.88). Furthermore, PwCC demonstrated increased healthcare service utilization, leading to a higher adjusted annual estimated mean cost (USD 4038 vs. USD 1833, *p* < 0.001). **Conclusions:** Chronic cough emerges as a relatively prevalent complaint within community care, exerting a considerable economic burden. This study underscores the need for heightened awareness, comprehensive management strategies, and resource allocation to address the multifaceted challenges associated with chronic cough.

## 1. Introduction

The cough is the most common symptom for which adults seek medical treatment in a non-hospital setting [1]. Among the myriad of manifestations of cough, chronic cough (CC), persisting for more than eight weeks [2], emerges as a prevalent condition with estimated prevalence rates ranging from 1% to 40%, dependent on population demographics and observational periods [3,4,5]. Beyond its prevalence, CC compromises patients’ overall quality of life, affecting physical, social, and psychological dimensions [6].

The complexity of CC is further compounded by its association with diverse underlying factors such as gastroesophageal reflux disease (GERD), postnasal drip from sinus infections or allergies, and various chronic lung conditions, including asthma, chronic obstructive pulmonary disease (COPD), bronchiectasis, pulmonary fibrosis, and interstitial lung diseases [7]. This multifaceted nature of CC contributes to its substantial impact, affecting up to 10% of individuals seeking medical assistance [8] and a staggering 46% of those referred to specialized cough clinics [9].

Despite the wealth of knowledge surrounding CC and the existence of guidelines for its diagnosis and management [10,11], a critical gap persists in the availability of population-based data encompassing demographics, clinical characteristics, diagnostic evaluations, treatment patterns, and healthcare service utilization (HCRU) related to CC. This scarcity, in part, can be attributed to the absence of specific International Classification of Diseases, Ninth Revision (ICD-9) or Tenth Revision (ICD-10) diagnostic codes for CC in many countries, including Israel, posing challenges to data retrieval.

In this study, we, therefore, aimed to assess the overall prevalence of CC and to determine the characteristics of patients with CC (PwCC) and their healthcare service utilization (HCRU) compared to a matched reference group.

## 2. Materials and Methods

### 2.1. Study Design

This retrospective cohort study was conducted using data from Maccabi Healthcare Services (MHS), a state-mandated health plan that provides healthcare for approximately one quarter of the Israeli population (circa 2.6 million members). Membership in MHS is free and open to all residents in Israel countrywide; with an annual membership retention rate of 99%.

### 2.2. Chronic-Cough Case Definition

**Figure 1:**
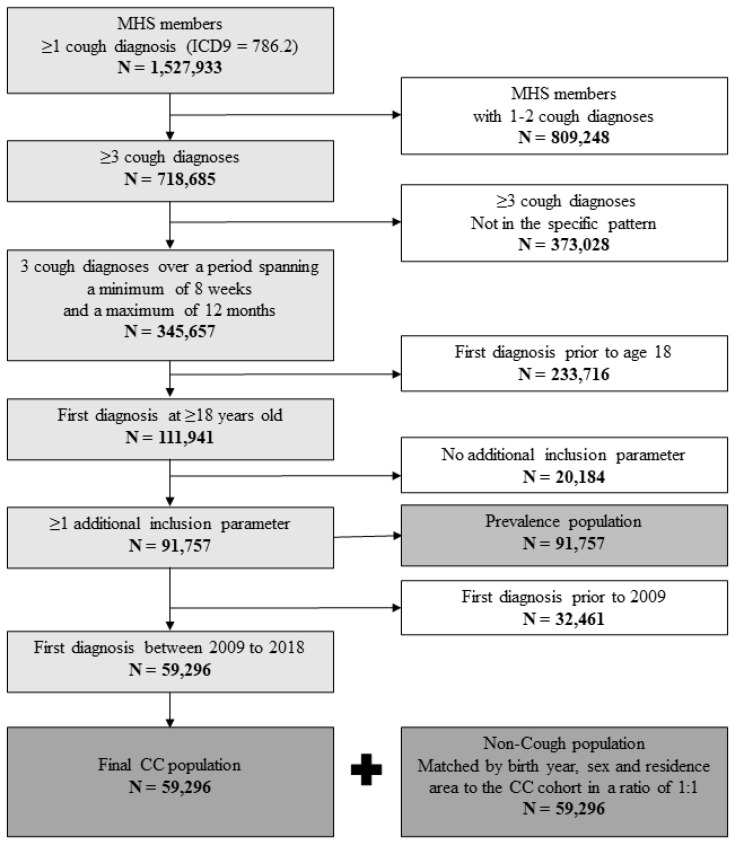
Identification algorithm for patients with chronic cough. MHS = Maccabi healthcare services, CC = chronic cough. bold = the emphasize the numbers, white = excluded, light grey = inclusion criteria, dark grey = final popualtions.

For this study, we considered patients aged 18 or above, with at least three cough encounters defined as physicians’ diagnoses of cough (ICD-9 code = 786.2) over a period spanning a minimum of 8 weeks. The maximal gap between diagnosis was 365 days anytime between 2009 and 2018 (Figure 1). The date of the first eligible cough encounter was defined as the index date.

The following supporting data were collected to support our case definition of PwCC: (a) visits to specialists that are likely related to CC (pulmonologists, gastroenterologists, ear-nose-throat [ENT], allergy, and occupational medicine); (b) performance of diagnostic tests suggestive of CC assessment (e.g., chest imaging, spirometry, see Supplementary Material [SM] file: Appendixes S1 and S2); and (c) prescription of medications specifically used to treat cough (e.g., antitussives, benzonatate, dextromethorphan, SM Appendix S3). The validity of our case definition was manually assessed via physician records’ free text evaluation by one researcher (YB) for CC description for a random sample of 250 patients. The algorithm had a positive predictive value of 72.8% (95%CI: 66.8–78.2%).

### 2.3. Reference Group

MHS members with no cough diagnosis in their electronic medical record (EMR) were matched by birth-year, sex, and residential area to the CC cohort in a 1:1 ratio and were defined as the reference group.

### 2.4. Other Study Variables

A measure of patient’s socioeconomic status (SES) on Points Location Intelligence^®^ group-level rank from 1 (lowest) to 10 was derived using the participants’ geocoded addresses. This score is highly correlated with SES measured by the Central Bureau of Statistics [12]. SES was categorized into low (1–4), medium (5–6), and high (7–10). Other characteristics such as height, weight, and smoking status within ±1 year were collected from the patient’s EMR.

History of co-morbid conditions that may have been potentially related to CC were defined according to ICD-9 including COPD, allergic rhinitis, respiratory tract infections, asthma, GERD, sinusitis, bronchiectasis, whooping cough, insomnia, fatigue, alcohol disorders, depression, and anxiety (SM Appendix S4). Comorbidities data at index date were obtained from MHS’s registries of cardiovascular disease registry [13], diabetes [14], hypertension [15], chronic kidney disease (CKD) [16], and osteoporosis [17]. We also collected information on cancer history including lung cancer as provided by the Israel cancer registry. All studied dispensed medications (acid suppression, asthma, cough, or nasal drip) during the first-year post-index date were documented.

### 2.5. Statistical Analyses

Descriptive statistics are presented as n, (%), or mean (±standard deviation [SD]) or median (interquartile range [IQR]), as appropriate. Life-time prevalence of documented CC was calculated for 2018. We also calculated the incidence and its Wilson Score 95% confidence interval (CI) based on the cases first documented during the 10-year study period.

For the comparison between PwCC and the reference cohort at baseline, standardized mean difference (SMD) is presented. An SMD that was less than 0.1 was taken to indicate a negligible difference in the means or prevalence of a covariate between groups [18]. Generalized linear regression modelling (GLM) with negative binomial distribution and log link function was used to model the frequency of hospitalizations, emergency department (ED) visits, and visits to primary care physicians (PCP), pulmonologist, gastroenterologist, allergy, ENT, occupational, and sleep specialists in the year post-index date. Negative binomial regression allows for analysis of count data when follow-up time differs between participants and because of overdispersion of the data. Cost analyses were performed for outpatient visits, hospital and ED visits, tests and procedures, medications, and overall cost. For cost analyses, gamma distribution with log link was used, after excluding the top 1% of the cost to exclude severe oncologic patients. Adjustments for baseline characteristics and comorbidities were made when needed. All analyses were conducted using IBM-SPSS Statistics for Windows, Version 27.0 (Armonk, NY, USA: IBM Corp), and a *p*-value < 0.05 was considered statistically significant. The study protocol was approved by the MHS intuitional review board (approval number: 0112-19-MHS given at 15 January 2020) and informed consent was waived.

## 3. Results

### 3.1. Identification of Chronic Cough Cohort

We identified a total of 91,757 patients who met the CC definitions (Figure 1). Of these, 59,296 patients (mean [SD] age, 53.9 [16.8] years; 59.6% females) were first diagnosed with CC during the 10-year study period, representing a 10-year period incidence rate of 3.26% (95%CI: 3.24–3.29%). A total of 84,057 PwCC were active MHS members in 2018 representing a prevalence rate of 5.5%.

PCP diagnosis accounted for 85% of the first diagnosing physicians. ENT and pulmonologists accounted for an additional 6.0% and 4.3%, respectively. A total of 13,730 (23.2%) and 15,143 (25.5%) had at least one cough diagnosis from ENT and pulmonologists, respectively.

The characteristics of the study population and the age-, sex-, and residential area-matched reference population are given in Table 1. PwCC were more likely (98.5%) to have a documented smoking status recorded in the EMR compared to the reference population (84.6%). Smoking rates among patients with available data among PwCC (14%) were comparable to the reference cohort.

### 3.2. Underlying Conditions in PwCC

**Figure 2:**
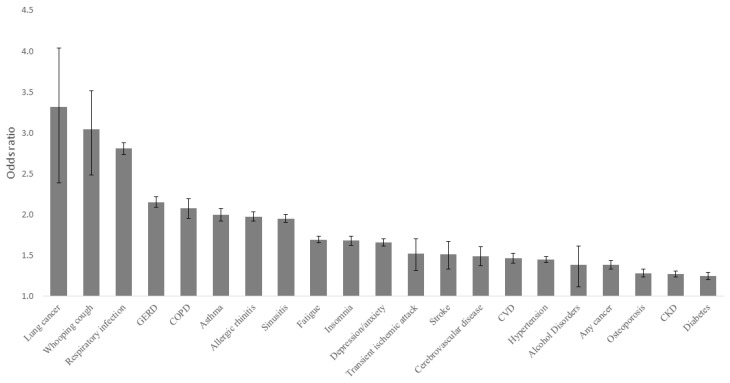
Odds ratio and 95% CI for chronic of underlying co-morbid conditions compared to a reference group matched for birth year, sex, and residential area. CI = Confidence interval.

The most frequent diagnosis among PwCC was respiratory infection (77.3%), substantially more frequent (SMD = 0.489) than in the reference cohort (54.8%). Other comorbidities with the largest differences in frequencies among PwCC compared to the reference cohort included sinusitis (36.2% vs. 22.6%), GERD (25.2% vs. 13.5%), allergic rhinitis (25.8% vs. 15.0%), fatigue (36.1% vs. 25.0%), depression and anxiety (34.0% vs. 23.7%), and asthma (14.2% vs. 7.7%), all with SMD > 0.2 (SM Appendix S5). Diseases associated with the highest OR for CC included lung cancer (OR = 3.32; 95%CI: 2.90–4.25), whooping cough (OR = 3.04; 95%CI: 2.70–3.60), and respiratory infections (OR = 2.81; 95%CI: 2.74–2.88), as depicted in Figure 2.

### 3.3. Use of Medications

A substantial proportion of PwCC were dispensed with at least one antitussive (81%) and nasal congestion medication (74%). These medications were first purchased during the one-year post-index date in 55.1% and 37.3% of patients, respectively. Other commonly dispensed drugs were acid suppression medications (48.0%) and asthma medications (43.4%) (Table 2). No medications of relevant classes were acquired in 3.4% of PwCC vs. 51% of the reference cohort.

### 3.4. Healthcare Resource Utilization and Related Cost

Compared with people without cough, PwCC had a significantly (*p* < 0.001) higher frequency of visits to pulmonologists and allergy specialists with adjusted odds ratios (ORs) of 12.05 (95%CI: 11.63–12.48) and 5.42 (95%CI: 5.12–5.74), respectively. PwCC were also more likely to visit other specialists as well as the hospital and ED, shown in Table 3.

The annual per patient adjusted estimated mean direct cost of the healthcare services utilized by PwCC (USD 4038) was significantly (*p* < 0.001) higher compared to people without cough (USD 1833), as shown in Table 4. The major drivers of the increased costs were physician visits and hospitalizations.

## 4. Discussion

In this study, we have used EMRs from a large population-based cohort to identify and characterize patients with CC. We provide information regarding the presence of co-morbid conditions, diagnostic evaluation, specialist consultations, and the use of cough-related medications in this population as compared to matched people without cough. Our data show that the presence of CC is associated with increased use of healthcare services.

The 5% prevalence of CC in our analysis was similar to a previous estimate from the Copenhagen General Population epidemiology study [19] and the 2018 National Health and Wellness Survey (NHWS) in the US [20], but lower than the global prevalence of 9.6% (7.6–11.7%) reported from a meta-analysis of 90 studies [21]. Most previous studies were based on self-reported questionnaires that included CC as part of a general inquiry into respiratory symptoms, while a recent analysis of administrative data showed an annual prevalence of approximately 1% [5]. It is very likely that not all PwCC seek medical attention and are, therefore, not documented in EMR. The database approach may be preferable to questionnaires, both because the accuracy of the diagnosis is better and because EMR visits reflect the perspective of health providers. The large heterogeneity in prevalence estimates can also be explained by the varying definitions and populations among reviewed studies (including 19 different definitions utilized in the meta-analysis) [21] and the lack of a specific diagnosis code. In addition, PwCC are frequently recorded according to the diagnosis of their underlying conditions such as asthma or GERD [21] and are not recorded with a diagnosis of CC.

The demographic characteristics of our CC cohort including older age and female predominance, were similar to those reported in previous studies [19,22] and to real-world data analyses [5,23,24,25]. In line with the results of previous epidemiological studies [2,19], GERD, asthma, and COPD were substantially more common among PwCC compared to people without cough [26]. In agreement with the Copenhagen General Population epidemiology study, PwCC had a two-fold higher prevalence risk of asthma and GERD compared to people without cough [19]. However, while the prevalence of asthma in PwCC vs. people without cough was comparable in the two studies, the frequency of GERD in our study was higher than in the Danish study in both PwCC (25% vs. 16%) and people without cough (13.5% vs. 8%). These findings indicate a greater comorbidity burden of GERD in the MHS population irrespective of CC.

Surprisingly, we found no difference in smoking rates amongst those with and without CC. This is in contrast to other studies in which smoking was an important cause or association with chronic cough in men [26] and in the entire cohort [21,27]. We do not have a clear explanation for this finding. However, female preponderance (59%) in our cohort may be one factor.

The negative impact of CC on quality of life has been observed in several population-based surveys [4,28,29] and is also associated with an increased likelihood of psychological conditions, including anxiety and depression [29,30]. In our study, more than one-third of PwCC had a documented diagnosis of depression or anxiety. Similar figures have been reported in several other studies [29,31,32].

In this cohort, we observed a high frequency of dispensing of respiratory and gastrointestinal medications that, in many cases, were first purchased in close proximity to the initial visit diagnosis of cough. However, we observed discrepancies between the number of patients with diagnoses of GERD or asthma and the number of patients using the respective medications, in line with previous publications [33,34,35]. These discrepancies might be a result of underdiagnoses of the above conditions, however, it is more likely they result of overuse outside of Food and Drug Administration indication [36,37].

In line with previous reports [28], PwCC were frequently referred to pulmonologists, ENT, and allergists, despite being initially diagnosed in primary care [20,38].

We found that the use of healthcare services among PwCC were significantly greater than in the reference group, not only with respect to medications and physician visits but also to hospital admissions and ED visits, in line with a previous study [29]. The adjusted mean direct medical cost of CC was approximately USD 4000 per patient, which is approximately 2.2 times higher than in the reference group. Nonetheless, we cannot exclude the possibility that the higher costs are a result of the additional, unmeasured comorbidities in the CC population. Especially in light of the greater number of ED visits and hospitalizations which are probably = not a direct result of the cough but rather a reflection of the impaired health state of the CC cohort. Higher estimates were calculated in the UK where the average direct cost of CC was GBP 3663 [39]. Our cost estimates do not include out-of-pocket expenses that are often incurred due to illness or indirect costs due to absenteeism from work. In the current analysis, costs for PwCC are primarily driven by physician visits, which likely includes the need for repeated evaluation by family physicians and possibly by specialist consultation. In addition, by definition, the CC cohort had a minimum of two additional visits unlike the control cohort. This may overestimate the difference in the healthcare resource utilization and costs between cohorts. However, this analysis found that the increase in costs for PwCC compared to those without cough was driven not only by outpatient visits, but from utilization of other healthcare resources as well.

As with any observational study, our study has some limitations. We did not assess the actual use of the dispensed medications. In addition, the observed utilization of healthcare services of the PwCC in the MHS system may not be generalizable to other populations. For example, referral rates in MHS where visits to PCP or specialists in MHS are free or require only minimal payment per visit differ from referral rates in other healthcare systems. Finally, in the scope of this study, we defined CC with a wide criteria, but were not able to describe the prevalence of refractory or unexplained CC.

In conclusion, CC is common amongst adults and characterized by increased use of healthcare services. Relative to people without cough, CC was also accompanied by an increased prevalence of depression and anxiety that may mark poorer quality of life. Further studies are needed in order to identify, characterize patients with different types of CC (i.e., refractory and unexplained CC), and assess their burden of disease.

## Figures and Tables

**Table 1:** Baseline year demographic characteristics of PwCC and people without cough matched by birth year, sex, and residential area.

		CC n = 59,296	Matched no Cough n = 59,296	SMD
Age at index, years	Mean (SD)	53.9 (16.8)	53.9 (16.8)	<0.001
Sex	Female (%)	35,358 (59.6%)	35,358 (59.6%)	<0.001
Residential area	North, (%)	11,335 (19.1%)	11,335 (19.1%)	<0.001
	Centre, (%)	37,720 (63.6%)	37,720 (63.6%)	
	South, (%)	10,241 (17.3%)	10,241 (17.3%)	
SES	Low, (%)	11,936 (20.1%)	11,325 (19.1%)	0.066
	Medium, (%)	21,411 (36.1%)	20,212 (34.1%)	
	High, (%)	25,824 (43.6%)	27,551 (46.5%)	
	Missing, (%)	125 (0.2%)	208 (0.4%)	
Weight, kg	Mean (SD)	77 (17)	75.8 (16.6)	0.068
Height, cm	Mean (SD)	165 (11.3)	164 (10.9)	0.002
Smoking status	Current, (%)	7914 (13.3%)	7036 (11.9%)	0.474
	Past, (%)	1761 (3.0%)	1484 (2.5%)	
	Never, (%)	48,757 (82.2%)	42,689 (72.0%)	
	Missing, (%)	864 (1.5%)	8087 (13.6%)	

PwCC = patients with chronic cough, SD = standard deviation, SES: socio-economic status, SMD = standardized mean difference.

**Table 2:** Dispensed medications of PwCC and people without cough during a one-year period the after index date ^a^.

	CC n = 59,296	Matched No Cough n = 59,296	SMD
**At Least One Purchase during the Year Post Index**			
Acid suppression medications	28,469 (48.0%)	12,945 (21.8%)	0.571
Asthma control medication	25,727 (43.4%)	4364 (7.4%)	0.91
Antitussive medications	47,921 (80.8%)	15,494 (26.1%)	1.311
Nasal congestion medications	44,010 (74.2%)	17,285 (29.2%)	1.011
**First Purchase during the Year Post Index**			
Acid suppression medications	10,717 (18.1%)	9670 (16.3%)	0.047
Asthma control medication	10,895 (18.4%)	2313 (3.9%)	0.473
Antitussive medications	32,645 (55.1%)	10,639 (17.9%)	0.835
Nasal congestion medications	22,095 (37.3%)	11,383 (19.2%)	0.41

PwCC = patients with chronic cough, SMD = standardized mean difference. ^a^ Percentages may add up to more than 100% due to multiple medication groups purchases per patient.

**Table 3:** Healthcare services utilization one-year post index-date among PwCC and people without cough.

	CC	Matched No Cough	Unadjusted	Adjusted ^a^	*p*-Value
			OR (95%CI) ^b^	OR (95%CI) ^b^	
Pulmonology visits	21,253 (35.8%)	1645 (2.8%)	13.16 (12.72–13.62)	12.05 (11.63–12.48)	<0.001
Allergy visits	5098 (8.6%)	708 (1.2%)	6.41 (6.06–6.78)	5.42 (5.12–5.74)	<0.001
ENT visits	27,655 (46.6%)	10,294 (17.4%)	3.33 (3.27–3.4)	2.73 (2.67–2.78)	<0.001
Sleep visits	1183 (2.0%)	416 (0.7%)	2.98 (2.72–3.26)	2.26 (2.06–2.49)	<0.001
PCP visits	59,111 (99.7%)	47,140 (79.5%)	2.22 (2.2–2.25)	2 (1.98–2.03)	<0.001
ED visits	9160 (15.4%)	4477 (7.6%)	2.38 (2.31–2.46)	1.94 (1.88–2.01)	<0.001
Gastroenterology visits	12,606 (21.3%)	6230 (10.5%)	2.19 (2.14–2.24)	1.76 (1.72–1.81)	<0.001
Hospital length of stay ^c^	11,026 (18.6%)	6382 (10.8%)	1.9 (1.87–1.93)	1.77 (1.73–1.8)	<0.001
Hospital admissions ^c^	11,026 (18.6%)	6382 (10.8%)	2.04 (1.99–2.1)	1.7 (1.65–1.75)	<0.001
Occupational physician visits	1795 (3.0%)	1009 (1.7%)	1.83 (1.72–1.94)	1.62 (1.53–1.73)	<0.001
Test and procedures	19,313 (32.6%)	3317 (5.6%)	1.42 (1.35–1.49)	1.42 (1.35–1.49)	<0.001

PwCC = patients with chronic cough, OR: odds ratio, PCP = primary care physicians, ENT = ear–nose–throat, ED = emergency department, CI = confidence interval. ^a^: Adjusted for smoking status, respiratory infection, hypertension, sinusitis, fatigue, depression and/or anxiety, allergic rhinitis, GERD, insomnia, asthma, CVD, COPD. ^b^: ORs and 95% CI were extracted from GLM with negative binomial distribution with log link. ^c^: For patients with at least one event.

**Table 4:** Healthcare costs ($US) in the year post index-date among PwCC and people without cough.

	Unadjusted			Adjusted ^a^		
	Estimated Mean (95%CI) ^b^		*p*-Value	Estimated Mean (95%CI) ^b^		*p*-Value
	PwCC	Matched No Cough		PwCC	Matched No Cough	
Visits	1327 (1315–1340)	587 (582–592)	<0.001	1992 (1941–2044)	945 (920–970)	<0.001
Hospital and ED ^c^	2726 (2660–2794)	2456 (2373–2543)	<0.001	5962 (5597–6351)	4853 (4526–5203)	<0.001
Test and procedures ^c^	216 (213–219)	197 (191–204)	<0.001	268 (258–278)	241 (230–253)	<0.001
Medications ^c^	85 (84–86)	55 (54–55)	<0.001	277 (269–285)	172 (167–177)	<0.001
Total costs	2095 (2074–2117)	885 (876–894)	<0.001	4038 (3922–4156)	1833 (1780–1888)	<0.001

PwCC = patients with chronic cough, CI = confidence interval. ^a^ Adjusted for Respiratory infection, hypertension, sinusitis, fatigue, depression and/or anxiety, allergic rhinitis, GERD, insomnia, asthma, CVD, and COPD. ^b^ Estimated means were extracted from GLM with Gamma distribution with log link. ^c^ The analyses for hospital and ED, test and procedures, and medications were performed for patients with at least one event. The top 1% of costs were excluded in each analysis.

## Data Availability

The study data included individual-level sensitive information. According to the regulation of the Israeli Ministry of Health (01/18) and MHS’s data privacy policy, patient-level data (including de-identified information) cannot be transferred outside MHS’s premise. Queries regarding the data can be addressed to: Maccabi Institute for Health Services Research. 4 Kaufmann St. Sharbat house, 8th floor, Tel Aviv, Israel Email: Barer_y@mac.org.il.

## Supplementary Materials

The following supporting information can be downloaded at: https://www.mdpi.com/article/10.3390/jcm13113230/s1, Appendix S1: List of lab tests; Appendix S2: List of procedures; Appendix S3: List of medications; Appendix S4: List of diagnoses; Appendix S5: Chronic co-morbid conditions among study groups.
