# Supplementary material for: Epidemiology and Healthcare Service Utilization among Adults with Chronic Cough
Source: J Clin Med. 2024 May 30;13(11):3230. doi: 10.3390/jcm13113230 (PMC11172840; doi:10.3390/jcm13113230)
Supplement: Supplementary file 1 [file jcm-13-03230-s001.zip › jcm-3026637-supplementary.pdf]

Supplementary material

Appendix S1: list of lab tests

Test	Description/CPT code
**Tuberculin**	Tubercul. Sputum Battery, Tuberculosis U Battery, Tuberculosis ource
**Sputum**	Eosinophil Sputum smear, Sputum Smear Battery XX, Monylia Sputum ulture, Sputum Culture Vitek, Sputum Epithelial Cells, Sputum Bacteria, Sputum Yeast Cult. Vitek, Mycobac.ID Sputum Culture, Sputum icroscopy, Sputum Culture, Sputum Yeast/Fungi Cult., Sputum Cult Polymo. Cells, Tubercul. Sputum Battery, Sputum Cytology, Mycobac. Sputum Culture, AFB Sputum Smear
**skin test**	994990023, 865800000, 865800001, 865800002
**Nose/throat smear dye**	891900000, 872050009

Appendix S2: list of procedures

Procedure/Test	CPT code
**Chest X ray**	742200000, 742400001, 742450001, 742450002, 742450003, 742450005, 757740001, 760000001, 764990004, 764990008, 710100002, 710100003, 710100005, 710100006, 710100008, 710100011, 710100012, 710100013, 710100014, 710100016, 710150001, 710150002, 710200000, 710230001, 710230002, 710600001, 516000001, 395990001, 395990002
**Chest CT**	763800004, 763800005, 763800006, 763800008, 763800011, 763800012, 763800018, 763800019, 763800020, 764970002, 712700001, 712750001
**Chest MRI**	715500009, 715510001, 715510002, 715550000, 715550001, 721960006, 715500000, 715500001, 715500008
**Esophageal manometery**	742300001, 910100000, 910340000, 782580001, 782620001, 433140003
**Spirometry and other pulmonary function tests**	940100000, 940100011, 940600005, 940600006, 785800000, 785850004, 785960000, 940100004, 940100007, 940100010, 940600001, 940600002, 940600004, 942400001
**Bronchoscopy**	710600001, 316150001, 316220002, 316220003, 316240000, 316250001, 316250002, 316350000, 316350001, 316350002, 316350004, 316360000, 316360001, 316360002, 316360003, 316410000
**Methacholin challenge**	940700001
**Upper gastrointestinal endoscopy**	432340001, 432340002, 432340003, 432340004, 432500001, 432500002, 432500003
**Laryngoscopy**	315050000, 315050001, 315050002, 315055100, 315250003, 315350002, 315450001, 315750000, 315760001

Appendix S3: list of medications

Medication	ATC code
**Cough preparations**	N03AX12, N03AX16, N06AA01, N06AA02, N06AA04, N06AA05, N06AA06, N06AA08, N06AA09, N06AA10, N06AA12, N06AA21, N06AB03, N06AB04, N06AB05, N06AB06, N06AB08, N06AB10, N06AF02, N06AF03, N06AF04, N06AG02, N06AX, N06AX03, N06AX05, N06AX06, N06AX11, N06AX12, N06AX16, N06AX17, N06AX18, N06AX21, N06AX26, N06AX27, R05CA02, R05CA03, R05CA10, R05CB01, R05CB02, R05CB03, R05CB13, R05CB15, R05DA04, R05DA09, R05DA20, R05DB01, R05DB07, R05DB23, R05FA02, R05FB01, R05FB02
**Nasal drip Meds**	R01BA52, R01AB01, R01BA02, R01AA07, R01AC01, R01AX03, R01AB, R01BA, R01AA, R01BA53, R01AA05, R01AD11, R01AX06, R01AC03, R01AD12, R01AC02, R01AD05, R06AX13, R06AD03, R06AD52, R06AB52, R06AX11, R06AE07, R06AX07, R06AD01, R06AD02, R06AA09, R06AX02, R06AX25, R06AB03, R06AX, R06AB, R06AK, R06AB04, R06AB54, R06AX12, R06AA52, R06AD08, R06AX17, R06AX15, R06AA08, R06AX27, R06AE51, R06AX26, R06AX09, R06AX29
**Asthma Meds**	R03AC03, R03AC02, R03AK07, R03AK11, R03AK10, R03AK08, R03AK06, R03AC13, R03AC05, R03AC18, R03AC12, R03AC19, R03AB02, R03AL03, R03AL06, R03AL04, R03AL05, R03BC01, R03BA05, R03BA01, R03BB04, R03BB01, R03BA02, R03BB07, R03BB06, R03BC03, R03BB05, R03DC03, R03DC01
**Acid Suppression Meds**	A02AA01, A02AX, A02BA03, A02AD01, A02BA02, A02BC03, A02BC05, A02AB10, A02AF02, A02AC01, A02BX03, A02BX12, A02BX13, A02BX05, A02BC02, A02BC06, A02BC01, A02BA01, A02AA03, A02AB01, A02AD04, A02BB01, A02AC10, A02BX02, A02AA04, A02BC01, A02BC02, A02BC03, A02BC05, A02BC06, A02BA03
**ACE-inhibitors**	C09AA09, C09AA02, C09AA01, C09AA05, C09AA03, C09AA07, C09AA08, C09AA06, C09AA13, C09BA05, C09BA08, C09BB02, C09BA09

Appendix S4: list of diagnoses

Comorbidity	Diagnosis
**Lung Cancer registry**	CANCER-RESPIRATORY-LUNG&BRONCHUS-UNSPEC.
	CANCER-RESPIRATORY-LUNG-NON SMALL CELL
	CANCER-RESPIRATORY-LUNG-SMALL CELL
	CANCER-RESPIRATORY-PLEURA
	CANCER-RESPIRATORY-TRACHEA
	CANCER-RESPIRATORY-UNSPECIFIED
**Whooping Cough**	ICD-9: 033*
**GERD**	ICD-9: 530.81
**Sleep apnea**	ICD-9: 780.5, 780.51, 780.53, 780.57, 327.2, 327.20, 327.21, 327.23, 327.27, 327.29
**Asthma**	ICD-9: 493*
**Allergic rhinitis**	ICD-9: 477*
**Respiratory Infection**	ICD-9: 465*, 466* , 490*, 491*, 492*, 493.2*, 494*, 496, 506, 506.4, 518.3 748.61
**Sinusitis**	ICD-9: 461.0, 461.1, 461.2, 461.3, 461.8, 461.9, 461, 473.0, 473.1, 473.2, 473.3, 473.8, 473.9, 473
**Insomnia**	ICD-9: 307.4*, 780.5*, 327, 327.0, 327.1*, 327.2*, 327.3*, 327.4*, 327.5*
**Depression + anxiety**	296.20, 296.22, 296.23, 296.30, 296.32, 296.33, 300.0*, 300.2*, 300.3, 300.4, 300.6, 300.7 , 300.8*, 308.3, 308.9, 300.9, 309.0, 309.1, 309.24, 309.28, 309.29, 309.3, 309.4, 309.8*, 309.9, 308.1, 308.2, 308.4, 311
**Fatigue**	780.7*
**Alcohol disorders**	305.0*

Appendix S5: Chronic co-morbid conditions among study groups

		CC<br>n = 59296	Matched<br>no cough<br>n = 59296	SMD
**Respiratory Infection**	n (%)	45822 (77.3%)	32480 (54.8%)	0.489
**Sinusitis**	n (%)	21485 (36.2%)	13377 (22.6%)	0.304
**GERD**	n (%)	14921 (25.2%)	8010 (13.5%)	0.298
**Allergic Rhinitis**	n (%)	15304 (25.8%)	8880 (15.0%)	0.271
**Fatigue, n (%)**	n (%)	21422 (36.1%)	14852 (25.0%)	0.242
**Depression and/or Anxiety**	n (%)	20145 (34.0%)	14039 (23.7%)	0.229
**Asthma**	n (%)	8442 (14.2%)	4554 (7.7%)	0.211
**Insomnia**	n (%)	11714 (19.8%)	7585 (12.8%)	0.189
**HTN**	n (%)	21875 (36.9%)	17070 (28.8%)	0.173
**COPD**	n (%)	3648 (6.2%)	1816 (3.1%)	0.148
**CVD**	n (%)	5941 (10.0%)	4193 (7.1%)	0.106
**Cancer**	n (%)	6752 (11.4%)	5045 (8.5%)	0.096
**CKD**	n (%)	12506 (21.1%)	10298 (17.4%)	0.095
**Whooping Cough**	n (%)	547 (0.9%)	181 (0.3%)	0.079
**Diabetes**	n (%)	8238 (13.9%)	6784 (11.4%)	0.074
**Osteoporosis**	n (%)	6513 (11.0%)	5210 (8.8%)	0.074
**Cerebrovascular Disease**	n (%)	1594 (2.7%)	1078 (1.8%)	0.059
**Lung Cancer**	n (%)	271 (0.5%)	82 (0.1%)	0.059
**Home Care**	n (%)	1112 (1.9%)	780 (1.3%)	0.045
**Stroke**	n (%)	775 (1.3%)	515 (0.9%)	0.042
**Transient ischemic attack**	n (%)	606 (1.0%)	400 (0.7%)	0.038
**Alcohol Disorders**	n (%)	281 (0.5%)	203 (0.3%)	0.021
